# Regional Internet Access and Mental Stress Among University Students: A Representative Nationwide Study of China

**DOI:** 10.3389/fpubh.2022.845978

**Published:** 2022-04-08

**Authors:** Shuhan Jiang, Weifang Zhang, Tingzhong Yang, Dan Wu, Lingwei Yu, Randall R. Cottrell

**Affiliations:** ^1^School of Humanities and Management, Zhejiang Chinese Medical University, Hangzhou, China; ^2^Key Laboratory of Oral Biomedical Research of Zhejiang Province, The Affiliated Stomatology Hospital, Zhejiang University School of Medicine, Hangzhou, China; ^3^Women's Hospital/Center for Tobacco Control Research, Zhejiang University, School of Medicine, Hangzhou, China; ^4^Injure Control Research Center, West Virginia University, Morgantown, WV, United States; ^5^School of Psychology/Center for Mental Health, Shenzhen University, Shenzhen, China; ^6^Center for Tobacco Control Research, Zhejiang University, School of Medicine, Hangzhou, China; ^7^Public Health Studies Program, University of North Carolina, Wilmington, NC, United States

**Keywords:** Internet use, mental health, mental stress, university students, China

## Abstract

**Background:**

The Internet changed the lives of average citizens in the early part of the twenty-first century, and it has now become an essential part of daily life. Many studies reported that accessibility of Internet use is associated with mental health. However, previous studies examining this association were confined to local and community subpopulations and limited at the individual level, which increases the potential bias from the selection effect at a different level. Regional variables would be a stable estimate of people's socioeconomic and cultural environments and how these variables affect mental health needed to be studied. The objective of this study was to evaluate the association between regional Internet access, and mental stress among university students.

**Methods:**

Participants were 11,954 students, who were identified through a multistage survey sampling process conducted in 50 Chinese universities. Regional Internet access was retrieved from a national database, and mental stress was measured using the Perceived Stress Scale (Chinese Version) (CPSS). Both unadjusted and adjusted methods were considered in the analyses.

**Results:**

More than one-third 36.9% (95% CI: 24.4–49.5%) of university students in this study suffered from severe mental stress (SMR). The multilevel logistic regression model found that university students studied in low-level universities had 2.52 (95% C.I. 1.17 to 6.37) times the prevalence of SMR than those in high-level universities. Compared with small cities, students in a large city had a lower prevalence of SMR (OR 0.25; 95%C.I. 0.06 to 0.77). Most importantly, regional Internet access was negatively associated with students' SMR (OR 0.25; 95%C.I. 0.08 to 0.76).

**Conclusions:**

This study indicated that regional Internet access and other environmental factors including city size and type of universities contribute to students' mental health. The findings underscore that efforts to control excessive mental stress among students in China should pay greater attention to environmental determinants of stress and particularly to improve internet access.

## Background

The number of Internet users has increased. An estimated 4.4 billion of the world's 7.7 billion people connected to the Internet in March 2019 (1). By 2008, China had outpaced the U.S. to become the world's largest Internet user (2). There are 1.01 billion Chinese Internet users and the Internet penetration rate reached 71.6% by June 2021 (3). The Internet changed the lives of average citizens in the early part of the twenty-first century, and it has now become an essential part of daily life. It is often used for such activities as shopping, obtaining health information, working, and communicating with each other. In the context of the global spread of Covid-19, the value and function of the Internet have once again been fully demonstrated.

The rapid growth of Internet uses around the world and in China—combined with the use of other Information and Communication Technologies (ICTs) such as personal computers and mobile devices—may have a significant impact on individual psychological well being (4). Many studies reported that fewer accessibility of Internet use is correlated with more psychological and mental problems, including depression, loneliness, and stress (5–7). And, Internet use would in fact buffer the mental and physical impact of stressful life events (8). Some studies, however, have found the opposite result: depression and psychosocial distress were positively related to Internet use (9–11). Regardless, the Internet has become an essential part of daily life and its impact on psychological and mental health needs to be closely studied. There is a rapidly growing public awareness of mental stress as an important factor that may lead to psychopathology (12).

According to the Stimulus, Cognition, and Response (SCR) model, various stimuli (S) affect internal states of people through cognition (C), which in turn elicits mental and behavioral responses (R). Classic S elicit different physical or mental outcomes due to personal cognition (13). In this context, maladaptive coping or cognitive styles may result in greater distress, while more positive personal lifestyles and access to social resources are associated with a more favorable psychological well being (14). Ecological models have emphasized that mental stress is influenced by both individual and environmental variables (15, 16). Access to and being able to use the Internet is a basic and important resource for people. Personal devices and behaviors affect access to the Internet, which in turn may affect people's mental stress (6). Regional variables are a widely accepted and stable estimate of people's behaviors (17). However, in reviewing the literature, previous studies examining Internet use and mental stress were confined to local and community subpopulations and limited at the individual level, which increases a potential selection effect bias (8, 10). Regional variables have been shown to be stable estimates of people's socioeconomic and cultural environments, so analysis results obtained from these variables are more reliable.

Mainland China is a vast territory with much cultural diversity and large differences in economic and social development. This creates a situation where different regions have vastly different levels of Internet access. Given China's regional differences in Internet access, it would seem that region of residence might also be related to mental stress, but no studies have directly examined this association. By utilizing a large-scale, national population sample in this study, it will be possible to expand and further clarify the relationship between regional Internet access and mental stress.

Numerous studies have reported that negative academic, emotional, and health outcomes may appear because of university students' high-stress levels (18–20). College students face multiple stressors including financial burdens, academic overload, constant pressure to succeed, competition with peers, and social pressure as well as concerns about the future (21). Besides, the majority of Chinese college students are the only child in their family. Lack of siblings, being spoiled and poorly developed psychological copying skills make them more vulnerable to mental problems (22).

A large-scale, national population sample will provide more representative information on the impact of regional factors including regional internet access on the mental stress of college students. This study hypothesized that higher regional Internet access will be associated with lower mental stress levels. The information from this study could be helpful to inform Internet development and access in China as well as mental health policy and intervention strategies for college students.

## Methods

This is a population study, the method used has been adopted by many researchers and proved to be reasonable and valid (23, 24).

### Study Area and Participants

This study employed a multistage sampling design. In Stage 1, 180 potential universities were identified in 45 China cities. These universities were part of the Bloomberg Global Initiative Project entitled “Facilitate MOH Endorsement of Tobacco Control Curriculum Implementation through Promoting Tobacco Control Curricula in Medical Schools.” Using a stratified random sampling procedure based on regional location, 60 universities were selected (25). All these universities were asked to participate and students from 50 of the 60 universities completed the study survey between March to September 2013. Among these 50 universities, 22 were medical universities offering only medical programs, and 28 were comprehensive universities offering medical and non-medical programs (26). Stage 2, of the sampling strategy, involved the selection of classes within each university. All classes with certain medical courses were selected in each university, and several non-medical classes (matched for academic level) were selected in each designated comprehensive university (25). A total of 11,954 valid questionnaires were completed in 50 universities including 10,507 completed by medical students and 1,447 completed by non-medical students. The study was approved by the Ethics Committee at the Zhejiang University Medical Center (ZM, 14201), and verbal consent was obtained from all participants before data collection.

### Measures

#### Dependent Variable

Mental stress was measured by the Chinese version of the Perceived Stress Scale (CPSS) (26–28). This scale comprised 14 items that addressed perceptions of stress during the month prior to the survey. Items were rated on a 5-point Likert-type scale that ranged from 0 (never) to 4 (very often). Item scores were summed to yield a total stress score, with higher scores indicating higher perceived levels of stress. This scale has been widely used to assess stress in China and has been shown to be an appropriate indicator of mental health status (28–31). Following previous practice, severe stress was operationalized as a score >25, which was classified by ROC (Receiver Operating Characteristic Curve) performance using mental disorders gold standard. This classification has demonstrated acceptable sensitivity and specificity (27). The dependent variable in this study was severe mental stress (SMS) and was coded dichotomously as 1 = no severe mental stress and 2 = severe mental stress.

#### Individual-Level Independent Variables

Sociodemographic questions were included to determine age, gender, grade level, ethnicity, paternal and maternal occupations, family location, and income.

#### Covariates

Injuries are one of the most common and prominent negative events among university students and questions regarding injuries were included in the questionnaire. Students were asked to identify any unintentional injuries which required medical attention during the past 12 months. Injuries were divided into the following four groups: traffic injuries, home injuries, sports or exercise injuries, and other injuries (32, 33). For the purpose of this study, a reportable injury was defined as any injury satisfying at least one of the following criteria: the injury required (1) a doctor's treatment (2) an emergency room visit or other emergency care, or (3) the victim to rest for a minimum of one-half day (32, 33). As a covariate, injury was a categorical variable coded dichotomously as 1 = yes and 0 = no.

#### University-Level Independent Variables

High levels of student stress may also reflect the type of institution where they are studying. University type was determined using the China university ranking system (“high level,” “middle level,” and “low level”) as established by the National Ministry of Education (34). Different level universities have different courses and opportunities, and levels of stress experienced by students were expected to vary in response to different learning environments (19). Due to the intense competition to enter elite universities, their higher tuition/fees, and elevated pressures to succeed, it was expected that stress levels would be highest at such institutions (35).

#### City-Level Independent Variable

Forty-five cities were included in this study. Several independent variables reflected potential regional variation. The first regional variable included in this study was the level of economic development, as measured by per capita Gross Domestic Product (GDP) in Yuan. Categories were <40,000, from 40,000 to <50,000, and 50,000 and more. Both the GDP of the original province where the students came from and of the province where the university was located were measured. Former research found that home region GDP was significantly associated with uncertainty stress (but not life stress) (23). Whether living in a college city different from the home city would affect mental stress was examined in this research. The above data were obtained from the National Bureau of Statistics (36). The second regional variable was regional Internet access status in cities where the university was located. This was measured by the number of subscribers to the broadband Internet per 10,000 persons, which was obtained from the National Bureau of Statistics (37). Categories were <40 subscribers of internet in 10,000 persons, from 40 to <70 subscribers of internet in 10,000 persons, and 70 and more subscribers of internet in 10,000 persons. Regional Internet access status reflected the extent of Internet use in each region.

### Data Analysis

All data were entered into a database using Microsoft Excel. The dataset was then imported into SAS (9.3 version) for statistical analyses. Descriptive statistics were calculated to determine the prevalence of severe mental stress. A logistic model was utilized to assess the association between the dependent variable and Internet access as well as several other key co-variables. Both unadjusted and adjusted methods were considered in the data analyses. SAS survey logistic procedures were applied in the unadjusted analysis, using the university as the clustering unit, to account for a within-clustering correlation attributable to the complex sample for unadjusted analysis. Associations were confirmed through the application of a multilevel logistic regression model using the SAS Nlmixed procedure (38). Series models were built for each primary predictor, with adjustment for the influence of potentially confounding sociodemographic characteristics. We started with the Null Model, a three-level (individual, universities, and cities) model with random intercepts. It did not include any predictors except a constant in assessing variation in the likelihood of an individual experiencing severe mental stress levels. From this model, we entered demographic and regional socioeconomic variables as fixed main effects with severe mental stress to form a base Model (Model 1). From the base Model, we entered Internet access status to form the full model to assess the impact of Regional Internet access on severe mental stress (Model 2). The association between system variables and mental stress was expressed in terms of their odds ratios, and 95% CI (Confidence Interval) was computed. Model fitting was assessed by the likelihood of a change in the −2log. We assessed the significance of the random parameter variance estimates using the Wald joint X2 test statistic (39). Model fit was assessed by the likelihood of a change in the – 2 log among different models.

All analysis was weighted. Weights included: (1) sampling weights, as the inverse of the probability of selection, calculated at university and (2) post-stratification weights, calculated in relation to sex, based on estimated distributions of this characteristic from a national survey (40). The final overall weights were computed as the product of the above two weights (41). Using a non-response weight was not considered because non-response rates were very low in this study.

Unadjusted logistic regression analyses were weighted using the overall participant-level weights, and the multilevel analysis was weighted using sampling weight and subject-level weights with post-stratification weights, respectively (42).

## Results

Valid questionnaires were completed by 11,954 out of 12,260 (97.5%) of the potential university students, who come from 50 different universities in the 42 cities.

### Demographics

Of those completing surveys, 12.8% were <20 years of age, 77.3% were between 20 and 23 years of age and 9.8% were over 23 years of age. Gender was split between 44.2 male and 55.8% female. The majority of responders (60.7%) were in their first or second years of study and, 38.5% were in their third or fourth years of study. Eighty-eight percent of respondents were medical students and 12% were from other majors (Table 1).

**Table 1:** Demographic characteristics of sample and mental stress prevalence.

**Group**	**N**	**%of sample**	**Prevalence**	**Unadjusted OR**
**Age (years)**				
<20	1,894	12.8	36.3	1.00
20-	2,392	32.3	34.9	0.91 (0.53, 1.56)
21-	2,762	30.6	44.5	1.38 (0.62, 3.01)
22-	2,450	14.4	34.4	0.93 (0.47, 1.82)
23-	2,456	9.8	26.7	0.64 (0.28, 1.45)
**Gender**				
Male	4,253	44.2	35.6	1.00
Female	7,701	55.8	38.1	1.12 (0.58, 2.16)
**Grade**				
1–2	4,945	60.7	36.0	1.00
3–4	6,717	38.5	39.5	1.16 (0.49, 2.74)
5–	292	0.8	17.0	0.39 (0.16, 0.90)^*^
**Ethnicity**				
Han	11,148	94.4	37.3.	1.00
Minority	806	4.2	35.1	0.90 (0.44, 1.85)
**Major**				
Medical	10,507	87.9	32.7	1.00
Others	1,447	12.1	38.1	1.26 (0.66, 2.41)
**Family home location**				
Rural or township	3,357	59.6	40.5	1.00
County town	769	17.2	35.6	0.81 (0.71, 1.16)
City	898	23.2	32.3	0.70 (0.47, 1.04)
**Income in each person in family (RMB)**				
<10,000	1,813	34.3	38.4	1.00
10,000	1,277	21.7	43.0	1.66 (0.85, 1.59)
20,000+	1,935	44.0	34.2	0.81 (0.64, 0.97)^*^
**Injure**				
No	6,889	51.5	36.0	1.00
Yes	5,065	48.5	38.4	1.11 (0.84, 1.45)
**University variables**				
**Universities types**				
High level	4,295	58.9	36.8	1.00
Middle level	6,961	39.5	36.1	0.93 (0.31, 2.82)
Low level	698	2.5	64.1	3.05 (1.45, 6.44)^**^
**Regional variables**				
**University city GDP**				
<50,000	4,055	16.1	31.4	1.00
50,000	6,378	61.1	38.0	1.31 (0.48, 3.59)
100.000	1, 521	22.8	39.0	1.38 (0.29, 6.67)
**City population (million)**				
<1	3,084	12.2	46.2	1.00
1–	5,982	57.3	42.4	0.85 (0.29, 2.45)
4–	2,888	30.5	23.7	0.35 (0.16, 0.77)^**^
**Number of subscribers of Internet/10,000 persons in university city**				
<40	4,394	53.5	57.9	1.00
40–	5,015	22.6	40.8	0.98 (0.27, 2.15)
70–	2,485	23.8	22.6	0.35 (0.14, 0.85)^**^

* *P < 0.05*;

** *P < 0.01*.

### Association Between Regional Internet and Mental Stress

**Figure 1:**
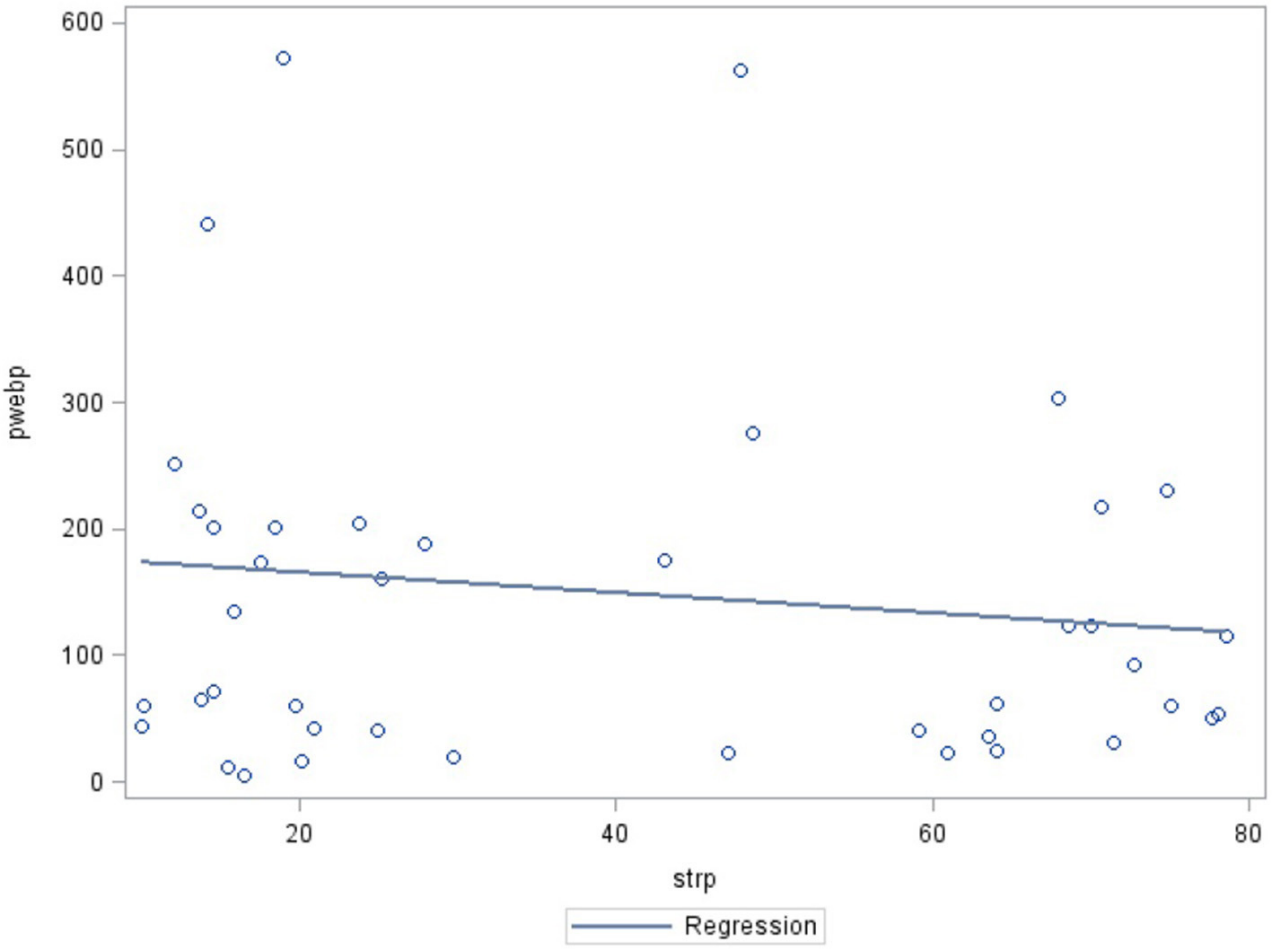
Relevant scatter plot between city-level number of subscribers of internet and severe mental afters prevalence.

The prevalence of severe mental stress among the study population was 36.9% (95% CI: 24.4–49.5%). To be considered having severe mental stress one had to score >25 on the stress scale. Lower Internet access was associated with higher mental stress prevalence. Scatter plot showing significant correlations between the city-level number of subscribers of the Internet and severe mental stress prevalence (R: −1.1296, *P* < 0.001). The lower the city-level number of Internet subscribers, the higher was the severe mental stress prevalence (Figure 1).

The results of two multiple-level regression models were listed in Table 2. The −2logs Likelihood of base model (model 1) is 32,849.3 and the −2logs Likelihood of full model (model 2) is 31,835.3. The comparison LRX2 is 14 (df = 1, *P* > 0.01) between them, suggesting that the effect of the full model fitting was well improved. In detail, the base model showed that grades, university type, and university city populations were significantly related to students' mental stress. The full model showed that regional variables, including types of universities, university city populations, and the number of website subscribers were significantly associated with the students' mental stress levels. Those studied in low-level universities had 2.52 (95% C.I. 1.17 to 6.37) times the prevalence of severe mental stress than the reference group. Small city students studying in a large city had a featured lower prevalence of severe mental stress (OR 0.25; 95%C.I. 0.06 to 0.77). Students in the regions with higher Internet access had less likelihood of having severe mental stress (OR 0.25; 95%C.I. 0.08 to 0.76) than those in the regions with lower Internet access (see Table 2).

**Table 2:** Results of multiple level models.

	**Base model**			**Full model**	
**Group**	**OR**	**95% C.I**.	***P***	**OR**	**95% C.I**.
**Grade**					
1–2	1.00			1.00	
3–4	1.23	0.52, 2.89	0.82	1.18	0.55, 2.54
5–	0.37	0.16, 0.86	0.02	0.41	0.19, 0.90^**^
**Type of universities**					
High level	1.00			1.00	
Middle level	0.93	0.31, 2.74	0.93	0.96	0.12, 2.44
Low level	3.21	1.61, 6.42	0.00	2.52	1.17, 6.37^**^
**University city population (million)**					
<1	1.00			1.00	
1–	0.85	0.31, 2.33	0.73	0.87	0.25, 3.05
4–	0.35	0.15, 0.77	0.01	0.25	0.06, 0.77^**^
**Number of subscribers of**					
**Internet/10,000 persons**					
<40				1.00	
40–				0.73	0.24, 2.21
70–				0.25	0.08, 0.76^**^
Fixed parameters	−0.18^*^				−0.27
Random parameters between universities	0.63^*^				0.58^*^
Random parameters between universities cities	0.57^**^				0.56^**^

* *P <0.05*;

** *P <0.01*.

## Discussion

Based on the results of this study, 36.9% (95% CI: 24.4 to 49.5) of university students were severely stressed, which is very similar to urban residents in general (36.8%) (95% CI: 33.5 to 40.2) (28). These results indicate that one-third of urban residents and one-third of university students are severely stressed. This mirrors findings from other countries. At Griffith University in Australia, fifty-three percent of first-year students were suffering from stress (41). In France, the mean perceived stress score among 1,876 students was 15.9. Scores between 16 and 20 were indicated stressed students (42). Mental stress is indeed a serious social and public issue that needs attention. Numerous studies have shown an escalation in the number of stress-related health problems in China (29, 43, 44). These high-stress levels have been attributed to massive social challenges such as an imbalance between urban and rural development, rampant corruption, and a widening chasm between rich and poor (14, 43, 45). The results of this study strongly suggest the importance of combating persistent and relatively high-stress levels among university students. The Central Government and local health authorities need to collaborate on policies for stress reduction and the prevention of mental disorders. Prevention needs to target those students who are at risk of severe stress. A nationwide media campaign should be planned and implemented to educate the populace about the adverse health effects of stress. Support groups should be established in urban community centers to provide a forum where residents can express mental health concerns, talk with others having similar concerns, and learn stress-reduction and management skills. Worksite programs should be established to help students manage their stress. University health authorities should offer stress management programs and concurrently offer mental health treatment as needed. Special university-based clinics should be established to provide high-risk individuals with no or low-cost psychological counseling on an as needed basis and in-patient care if required. Although traditional face to face cognitive behavioral therapy and stress management program are generally recognized as great sets of stress reduction (46, 47), the obvious characteristics of those approaches are rigorous, time-consuming, and costly, which may not be ideal for college students (48). Previous studies reported that internet-based and mobile-based programs focusing on mindfulness meditation and positive psychology interventions can be effective in improving students' stress outcomes (48–50).

Addressing a gap in the literature, our study found regional Internet access was associated with students' mental stress. Based on running full analysis models, people in the regions with lower Internet access had 4 times the likelihood of having severe mental stress than those in the regions with higher Internet access, and this result was consistent with the analyses using the mental stress score as continuous or categorical data. The mean mental stress scores in the three different city-level internet access groups which can be seen in Table 1. There were significant differences among the three groups. When the variables listed in Table 1 were controlled by multivariate stepwise regression, the regression coefficient of the relationship between city-level Internet access and psychological stress scores was 0.03 (*P* < 0.001). Internet access reflected the number of Internet subscribers among residents in these regions (2). For this study, Internet access was considered as an essential regional variable separate from general socioeconomic characteristics. It has been noted that “inequalities in access to and use of Internet has become one of the most prominent forms of social inequality with a major influence on life opportunities” (48, 51). Several studies have linked such a “digital divide” to poor health and well being (52–54). College students who live in a city with lower Internet access could be considered at a disadvantage in terms of their overall learning and their competitiveness in the job market.

Moreover, in modern society, the Internet has become an essential part of daily life, for shopping, obtaining information, working, and communicating. On the one hand, Internet access is the basis for the everyday social life of long-distance communication and social participation. Involvement in varied kinds of online social networking, on the one hand, can contribute to feelings of self-worth and, on the other hand, can provide more solutions for distressed people, especially when traditional resources are not available or affordable (55). People who cannot conduct these activities due to lack of Internet access could feel severe psychological discomfort which may lead to mental disorders. Besides, it should be noted that the negative impacts of the Internet, digital products, and mobile devices emerged among university students with the rapid development of internet technology (56–58). Further research into the relationship between individual Internet use patterns and physical and mental development among young adults is necessary.

Regional Internet access status is dependent on urban economic progress; the survey showed that higher Internet access regions were in economically developed regions, especially the eastern coastal areas of China (59). At the same time, socioeconomic status is also associated with mental stress (60). It is possible that regional social and economic variables may play a confounding role in the association between regional Internet access and students' mental stress. Our study controlled for the potential interference of regional social and economic variables.

Unlike other studies (61, 62), this study found other environmental and regional variables were also associated with severe mental stress. Firstly, the city size where universities were located was associated with students' mental stress. Large cities in China usually have more financial resources and technology available to students. They also have better social services (37). This reality may mean that students in large cities have less mental stress than students in small cities. This study also revealed that students attending higher-level universities have lower mental stress prevalence than students attending lower-level universities. It is plausible that higher-level universities attract more outstanding students. Students attending higher-level universities have access to better facilities and learning resources and may face less employment pressure as they are more likely to be employed upon graduation than students attending lower-level universities (19). Ultimately the advantages of attending a higher-level university may relieve some of the stressors associated with being a college student.

The results of this study indicate that environmental conditions are important contributors to university students' mental health. To promote students' mental health, it is important to address negative environmental conditions and develop programs to help students manage their stress levels.

### Study Limitations

Several limitations to this study must be considered when interpreting the results. The cross-sectional study design is an important limitation of our study; therefore, a causal link between regional Internet access and mental stress cannot be established through this work. On the other hand, we employed a large sample, and our findings met several criteria for inferring causality, including the strength of some associations, their consistency, and plausibility of effect. Future studies need to collect longitudinal surveillance data on the mental health of college students, especially focusing on the relationship between individual Internet use patterns and mental stress in the context of the influence of regional variables. Another important limitation is that our participants were confined to university students, particularly medical students. Thus, our results cannot be generalized to the wider Chinese population.

## Conclusion

This study indicated that regional Internet access and other environmental factors including city size and type of universities contribute to students' mental stress. The findings underscore that efforts to control excessive mental stress among college students in China should pay greater attention to environmental determinants of stress and particularly to improve internet access.

## Data Availability Statement

The raw data supporting the conclusions of this article will be made available by the authors, without undue reservation.

## Ethics Statement

Written informed consent was obtained from the individual(s) for the publication of any potentially identifiable images or data included in this article.

## Author Contributions

TY conceived the study. SJ drafted the first versions of the manuscript. WZ and RC revised the manuscript. DW and LY collected the data. All authors contributed to the article and approved the submitted version.

## Funding

This study was partly funded by the National Nature Science Foundation of China (71490733) and the Nature Science Foundation of Zhejiang Province (LQ20G030014).

## Conflict of Interest

The authors declare that the research was conducted in the absence of any commercial or financial relationships that could be construed as a potential conflict of interest.

## Publisher's Note

All claims expressed in this article are solely those of the authors and do not necessarily represent those of their affiliated organizations, or those of the publisher, the editors and the reviewers. Any product that may be evaluated in this article, or claim that may be made by its manufacturer, is not guaranteed or endorsed by the publisher.

## Abbreviations

CPSS: the chinese version of the perceived stress scale
GDP: gross domestic product
SMS: severe mental stress.
